# Factor V Leiden and Inflammation

**DOI:** 10.1155/2012/594986

**Published:** 2012-05-14

**Authors:** Silvia Perez-Pujol, Omer Aras, Gines Escolar

**Affiliations:** ^1^Servicio de Hemoterapia y Hemostasia, Hospital Clínic, C/Villarroel 170, 08036 Barcelona, Spain; ^2^Molecular Imaging Program, National Cancer Institute, 10 Center Drive, Building 10, Room B3B85, Bethesda, MD 20892, USA

## Abstract

Factor V Leiden, is a variant of human factor V (FV), also known as proaccelerin, which leads to a hypercoagulable state. Along these years, factor V Leiden (FVL) has been studied from the pathophysiologic point of view, and research has been focused on finding clinical approaches for the management of the FVL associated to a trombophilic state. Less attention has been paid about the possible role of FVL in inflammatory conditions known to be present in different disorders such as uremia, cirrhosis, liver transplantation, depression as well as sepsis, infection or, inflammatory bowel disease (IBD). Whether platelet FVL will increase the activation of coagulation and/or in which proportion is able to determine the final outcome in the previously mentioned inflammatory conditions is a subject that remains uncertain. This paper will review the association of FVL with inflammation. Specifically, it will analyze the important role of the endothelium and the contribution of other inflammatory components involved at both the immune and vascular levels. This paper will also try to emphasize the importance of being a FVL carrier in associations to diseases where a chronic inflammation occurs, and how this condition may be determinant in the progression and outcome of a specific clinic situation.

## 1. Introduction

Coagulation factor V (FV), also known as proaccelerin or labile factor, is synthesized in the liver and circulates as a single chain polypeptide in an inactive procoagulant form [1]. Although most FV is present in plasma, approximately 20% of the circulating FV is found within *α*-granules [2]. Platelet FV is partially proteolysed and is stored bound to the protein multimerin inside those granules [3]. Factor V plays a key role as a cofactor of the prothrombinase complex that cleaves and activates prothrombin to thrombin leading the formation of the blood clot [4]. Factor V Leiden (FVL) is the name of a specific gene mutation which leads to a hypercoagulability state with serious clinical consequences [5]. Less than two decades of its discovery, the pathophysiology, clinical consequences, and therapeutic management of the thrombophilic state associated to this condition remains object of controversies [6]. The excessive clotting that occurs in this FV disorder is mostly restricted to the venous territory and it's known to be the most frequent contributors to cause deep vein thrombosis (DVT) [7, 8].

Sometimes venous clots may break off and travel through the heart and lung blocking vessels causing pulmonary embolisms. On the other hand, although seems less common the formation of clots in arteries, they may lead to stroke or myocardial infarction (ministroke or transient ischemic attacks) [9]. Interestingly, in the last years, major attention is being paid on how venous thromboembolism (VTE) is becoming a serious public health problem contributing to a steadily increasing mortality in our modern societies [10, 11].

It is well known that venous thrombosis (VT) occurs as a result of a combination of genetic and environmental risk factors such as age, surgery, trauma, cancer, pregnancy, and use of hormonal therapy. The presence and interaction of one or more of those risk factors will determine the relative risk of developing VT. FVL mutation will further enhance the risk of developing abnormal clots, being currently considered the most common cause of Thrombophilia [10].

A more limited information is available on the impact of FVL in clinical events where inflammation is prevalent [12, 13]. Uremia, cirrhosis, liver transplantation, but also sepsis (generalized inflammatory state also known as SIR-systemic inflammatory response), infection, and Inflammatory Bowel Disease (IBD) result in a chronic inflammatory state. Even though, both mechanisms of action and clinical management of the previous conditions are well established, it has not been possible to reach a final consensus on whether FVL is able to modify the outcome of these events.

The inflammatory response is a complex reaction which implies the interaction of vascular tissues, coagulation mechanisms, and blood cell components, mainly platelets (Plts) and leukocytes [14, 15]. Platelets are the main blood “cell” element involved in the formation of the clot [16]. Attachment to the subendothelium, activation with release of its granule content, and aggregation provide the appropriate environment for the activation of the coagulation mechanisms [17, 18]. During this process, FV, released upon platelet activation, plays a crucial role in the early phase of the coagulation expressed in its an active form (FVa) for immediate and sustained procoagulant function at sites of vessel injury. Leukocytes and cytokines they produce, are critically involved in initiating and maintaining the inflammatory response. These cells will be recruited and attracted at the site of injury through selectins which became exposed on inflammated endothelium or on activated platelets.

In past years, researchers have been mainly focused on the insights of the immune and coagulation systems during the inflammatory response, but less attention has been paid to the role of the damaged endothelium, activated Plts, and circulating microparticles (MPs) potentiating the abnormal functionality of FVL. Available literature on the possible relation among FV mutations, platelet function, and increased number of circulating MPs is relatively scarce [19].

Through the following text, we will review the association of FVL with inflammation as well as provide new insights/other aspects, which may contribute to a better understanding of specific diseases, which undergo chronic inflammation including sepsis, infection, and/or IBD. Special attention will be paid to the contribution of the endothelium, activated platelets and derived MPs as carriers not only of FV, but of Tissue Factor (TF) during inflammatory conditions and its possible implications in thrombotic complications in patients diagnosed with FVL.

## 2. Pathophysiology of Factor V: Factor V Leiden Mutation

Blood clots form in two steps. In the first step, activated Plts release the content of stored granules into the blood plasma (primary Hemostasis) and aggregate forming an initial plug or clot. In a second step, Plts attract more Plts and coagulation factors become activated [20]. Overall, Plts are associated together with the coagulation proteins and contribute to fibrin generation that will stabilize the blood clot [17].

**Figure 1:**
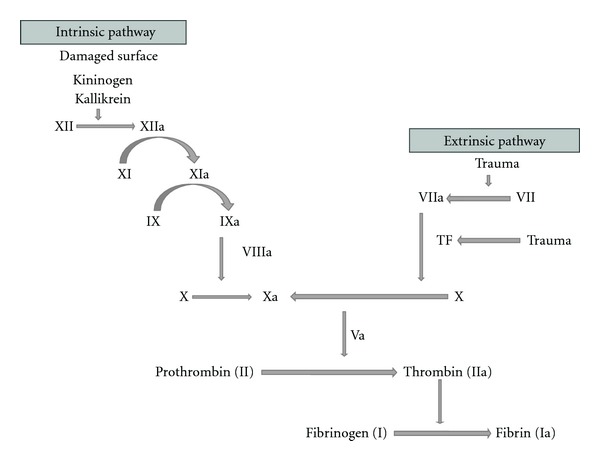
Coagulation cascade.

**Figure 2:**
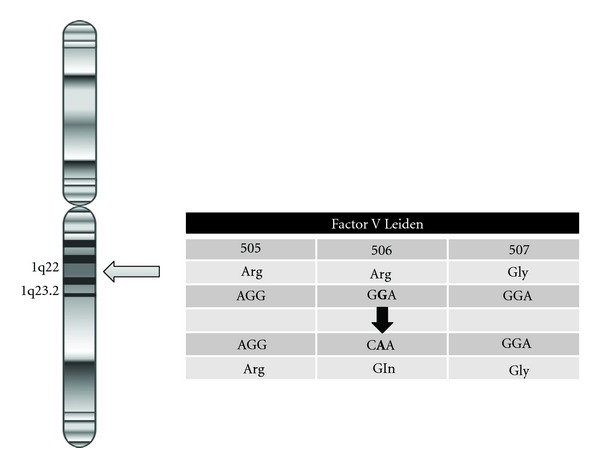
FV gene. The FV gene is located on the long arm (q arm) of chromosome 1 at position 23. This mutation results of a single change in one aminoacid (single nucleotide polymorphism; SNP) in the factor V protien. Specifically an arginine is replaced by a glutamine at protein position 506 (Arg506Gln or R506Q).

Factor V protein circulates in the bloodstream as an inactive form until the coagulation system is activated due to a damage in blood vessels. In a normal state, activated FV (FVa) acts as a cofactor that interacts with active Factor X to convert the inactive enzyme prothrombin into thrombin which cleaves Fibrinogen to Fibrin [4]. Then, Fibrin polymerizes leading to the formation of the blood clot (see Figure 1). Participation of activated protein C (APC) as a natural anticoagulant limits the extent of clotting by cleaving and degrading FV [21]. Activated protein C (APC) cleaves FV at specific sites (at protein position 506) and downregulates its function therefore preventing the excessive growth of the clot [3, 22]. Cleavage of FV will also allow this coagulation factor to inactivate factor VIIIa, another essential protein for the normal blood clotting process [23–25]. APC is unable to inactivate FV when the mutation on this coagulation protein occurs: single change of one aminoacid at position 506; an arginine (Arg) is replaced by a glutamine (Gln) (see Figure 2) [26]. As a result of this, the mutation leads to a loss of anticoagulant cofactor function of APC on FV with clotting mechanisms remaining active for more prolonged periods and a subsequent increase of the formation of pathological blood clots. This is the reason why FVL disorder was initially described as APC resistance, although the FVL mutation is only found in about 80–90% of cases diagnosed with this condition [6, 27, 28].

The FVL mutation is currently the most common known risk factor for VT. Factor V Leiden is an autosomal dominant condition only present in Caucasians, and the prevalence varies between 2% to 15% [29]. It has been demonstrated that the mutation is less common in other populations such as Hispanics and African-Americans, and it's extremely rare in people of Asian descent [30, 31]. The polymorphism occurred from a common source about 30 000 years ago (founder effect) and between 3–8% of people with European ancestry and 5% of Caucasians in North America carry one copy of FVL mutation, meanwhile about 1% (5,000 people approximately) has two copies of the mutation [32].

The presence of the FVL mutation considerably increases the risk of developing abnormal blood clotting. The most common clinical manifestations of FVL are superficial and DVT. Actually, up to 30% of patients who presents DVT has been diagnosed with this condition [10]. The risk of developing thrombotic episodes depends on whether a person has one or two copies of the FVL mutation. Inheritance of two copies of the mutation, one from each parent, significantly increases the risk of developing thrombotic complications [33]. Overall, those who are homozygous for the mutated allele present an increasing risk of developing abnormal clots (50–100 fold) versus those that are heterozygous for the mutation (5–10 fold) who actually present a quite low risk for venous thrombosis [31]. It is important to consider that the combined association of another acquired risk factors for VT such as age, obesity, smoking, use of birth control pills (estrogen based hormonal contraception), or hormone replacement therapy recent surgery, will further enhance the risk for an individual carrying with the FVL mutation to develop DVT [34]. Furthermore, the association of the FVL mutation with another mutation in another gene involved in the coagulation system will cause an extremely high risk of developing abnormal clots. In this context, coexistence of heterozygous FVL mutation and the G20210A mutation in the prothrombin gene is the most frequent abnormality causing VTE [35, 36]. Moreover, combinations of FVL, antithrombin, protein C or protein S deficiencies and hyperhomocystienemia, have been found to result in a 5-to-10 fold increase in the relative risk of thrombosis (see Table 1-adapted from “Natural anticoagulants and thrombophilia”) [37, 38].

Although FV synthesis was previously determined to take place in megakaryocytes [39], Christiella et al. [25] were able to demonstrate that plasma is the principal origin for platelet FV. However, a small proportion of FV is found within the platelet *α*-granules. Gould et al. [3] provided a strong evidence that once FV stored in *α*-granules is a different substrate. Once being endocytosed by megakaryocyte and delivered to Plts, FV is cleaved to a partially active cofactor, which appears to be more resistant to APC inactivation. This finding supported the research of Camire et al. [40] demonstrating that complete inactivation of platelet-derived FV was never accomplished. According to the previous studies, FVa that expressed on activated Plts could be protected from inactivation by APC. Further experiments also demonstrated that the platelet-derived MPs resulted in increased levels/rates of the cofactor inactivation suppressing the apparent protection developed by intact Plts [41]. This is an important point to take into consideration since elevated levels of circulating MPs have been reported in patients diagnosed with FVL [19]. Further studies have demonstrated that levels of MPs may correlate with plasma levels of FVL thus suggesting that the ratio FVL/FV ratio could be a parameter to predict the risk of thrombosis in carriers of FVL [42].

## 3. Pathophysiology of Inflammation: Role of Endothelial Dysfunction, Platelets, and MPs

Inflammation can be defined as a complex biological response of protection to a wide variety of harmful stimulus. This inflammatory response undergoes with a break of the balance of the coagulation and alterations at multiple levels involving damage at vascular tissues, reaction of leukocytes releasing interleukins and cytokines, and activation of platelets [15, 43]. Inflammation is classified as acute or chronic. Acute inflammation is the initial response of the body leading to a cascade of biochemical events based on the interaction of the vascular and immune system. Persistent or chronic inflammation results in elevation of several procoagulant proteins such as fibrinogen and Factor VIII that have been considered as contributors to the development of a thrombophilic state [38]. Chronic inflammatory response may lead to a disruption of the procoagulant/anticoagulant hemostatic equilibrium together with other alterations at multiple levels. Briefly, there is a reaction of blood cells, mainly leukocytes, which release interleukins and cytokines that will perpetuate the inflammatory response [43, 44].

Endothelial dysfunction is almost a constant in the inflammatory process [45]. As a result of the inflammatory response, the permeability of the blood vessels increase and recruitment of leukocytes at the site of injury is initiated. Basically, endothelial disruption causes the increased expression of new adhesion molecules such as E-selectin, PECAM-1 (platelet-endothelial cell adhesion molecule 1), VCAM-1 (vascular cell adhesion protein 1), ICAM-1 (Inter-cellular adhesion molecule 1), endothelin-1, release of Von Willebrand factor (vWF), and P-selectin and L-selectin from storage granules known as Weibel-Palade bodies [46, 47]. Endothelial-selectin expressions are essential for leukocyte rolling and infiltration into the vessel wall [48]. Moreover, exposure of TF on the endothelial surface, which is upregulated by multiple inflammatory proteins, activates the coagulation cascade and promotes the procoagulant activity of Plts [49–51]. All these changes facilitate the adhesion and activation of Plts to the damaged endothelium establishing a favorable environment for thrombus formation [18, 52, 53]. Alterations caused by the endothelial dysfunction are aggravated by the fact that during prolonged inflammation, both mechanisms of nitric oxide and prostacyclin production are also impaired promoting the adhesion of the Plts to endothelium [45].

Formation of MPs has also revealed an important event for the inflammatory progression [54, 55]. Microparticles are small portions of membrane shed from activated cells such as endothelial, monocytes, and/or platelets which support procoagulant activity and contribute to the formation of the hemostatic plug [41, 56–58]. In 2004, Lopez et al. [45, 59] proposed a model based on monocytes/macrophages derived microvesicles (MVs) bearing TF with the ability to fuse with activated endothelial cells due to inflammation and promote the initiation of blood coagulation. Therefore, endothelial-derived adhesion molecules, specifically P- and E-selectin would play a key role interacting with platelets and circulating MVs/MPs bearing TF. Transfer of TF to the endothelial cells will promote the procoagulant activity, enhancing the thrombin generation and the formation of the clot [60–62]. The contribution of circulating MPs to inflammation was shown again when Salanova et al. [63] demonstrated that platelet GPIIb-IIIa receptor is acquired by neutrophils via Plt-derived MPs leading the activation of NF-*κβ* signaling cascade supporting the inflammatory response [64].

A recent publication has reviewed the important role of Plts and Plts-derived MPs not only in hemostasis and coagulation but in some other diseases including: atherosclerosis and related diseases, pathologies of the central nervous system (Alzheimer's disease, Multiple Sclerosis), and even cancer and/or Tumour growth [65]. Thus, Plts are essential for the innate immune response, combat infections (viruses, bacteria, and microorganisms) and develop an important role during the inflammatory processes [66].

## 4. Factor V Leiden and Inflammation: Infection, Sepsis, and Inflammatory Bowel Disease

During all these years, research in FVL polymorphisms has been centered on determining the risk of possible thrombotic complications in different clinical situations (surgery, pregnancy) and evaluating the risk/benefit of prophylactic treatments. Less attention has been paid to the possible association of FVL with inflammation [67]. Within past 5 years, some researchers have started to question whether FVL may be a key condition determining the outcome of clinical episodes such as infection, sepsis and/or IBD [68]. Less has been discussed about the possible effect of the Plts and platelet-derived MPs as a carriers of a modified FVL and in which level they may be able to modulate the outcome of this process associated to sepsis, infection, or/and IBD [42].

### 4.1. Infection and Sepsis

Infection and severe sepsis imply an inflammatory process with an important alteration in their coagulation system [49, 69]. In the majority of cases, sepsis is usually caused by the presence of a known or suspected infection. In septic patients, all three of the classic Virshow's triad are present and culminate in compromised blood flow to vital organs causing a major organ failure. The coagulation abnormalities in septic patients are profound and life threatening [70]. In 2004, Weiler et al. [71] tried to demonstrate that the polymorphism in FVL was able to modify sepsis outcome according to animal models. However, further studies could not find a significant correlation between FVL and some other hemostatic polymorphisms as predictors of prognosis of sepsis [72–74].

In 2008, Lindqvist and Dahlbäck [75] indicated the possibility of FVL being an evolutionary advantage against specific events such as sepsis based on the high prevalence in the general population. In the same line, Isma et al. [76] also supported that thrombotic events may be considered a survival advantage against specific physiological conditions, specifically in women. In contrast to this idea, numerous publications revealed that the risk of suffering miscarriage during pregnancy in women carrying FVL is slightly increased (two or three upfold) as well as presenting complications derived of clotting episodes during pregnancy such as suffering DVT, pulmonary embolism, preeclampsia, slow fetal growth, and/or early separation of the placenta from the uterine wall (placental abruption) [77, 78]. Concentrates of APC have been considered to have a beneficial effect when associated to the standard therapy (antibiotics, fluids, and blood components) for sepsis. Besides its anti-thrombin effect, APC holds anti-inflammatory action due to its interaction with white blood cells and endothelial cells [79–81]. Since Plts and MPs are carriers of a FVL modified which becomes resistant to the inactivation of APC, it would be interesting to evaluate to which extent the APC treatment is equally effective in carriers of FVL or even if it will be able to influence an earlier or delayed response to the standard treatment.

As in sepsis, publications are also controversial about the influence of FVL mutation in the outcome of infections such as pneumonia or flu [70]. Meanwhile, Schouten et al. [82] were able to find some differences in the outcome of pneumococcal pneumonia, further studies revealed that no significant changes were found in the outcome of infection by lethal H1N1 influenza.

Various publications have revealed that abundant circulating MPs are present during acute infections such as in plasmodium vivax infection. This finding supports the idea that not only Plts but also platelet derived-MPs may play a role on the acute inflammatory symptoms which occur during infections [83].

### 4.2. Inflammatory Bowel Disease

Inflammatory bowel disease (IBD) is known as a group of inflammatory conditions of the colon and small intestine and usually, it refers to two chronic diseases: ulcerative colitis and Crohn's disease. A hypercoagulable state has been recognized in patients with IBD. In fact, patients suffering from IBD have about threefold increased risk of VT [84, 85].

Numerous studies have been performed in an attempt to detect a relationship with the presence of FVL mutation and the incidence of IBD [86]. The majority of publications have not found a clear association between FVL and the risk of developing IBD, although FVL continues to be considered a risk factor which may influence the clinical manifestations during IBD [84, 86–88]. Similarly, Liang et al. [89] revealed that FVL mutation was not significantly associated with the risk of developing IBD. In contrast with the previous reports, studies by Magro et al. [90] support the concept of higher prevalence of single and combined thrombophilic defects in IBD patients, stating that these factors may be involved in the disease pathogenesis. There is no final conclusion about the possible role of FVL mutation but seems clear that presence of FVL mutation in patients with IBD increases the risk of developing thrombotic episodes. The pathophysiology of IBD is still under active investigation. The inflammatory process in IBD is probably associated to an immune reaction of the body against its own vascular tissue [91]. As a result of this damage, leukocytes and Plts become activated and trigger a chain reaction causing the complete coagulation cascade dysregulation promoting the formation of clots [92, 93].

Interestingly, elevated levels of circulating MPs have been found in patients with active IBD. More specifically, Andoh et al. [94] were able to determine the increased reaction of Plts in patients suffering IBD having an important role in its pathogenesis. Immune system reaction against the own vasculature may generate increased circulating levels of MPs, which may play a role in enhancing coagulation and inflammatory states in these patients. Leonetti et al. [95] demonstrated, using an experimental model, that platelet-derived MPs influence and improve both endothelial dysfunction and vascular hyporeactivity promoting the thrombophilic state, which will be probably enhanced in those patients diagnosed with FVL [54, 96].

## 5. Final Considerations

Thrombophilic state arises from the interaction of a number of different factors including hereditary deficiencies such as FVL, acquired deficiencies in natural anticoagulants and dysfunctional coagulation factors with a precipitating clinical situation [97]. Despite of being considered clinically different, an inflammation response is a common early phenomenon, which occurs in numerous clinical situations including those mentioned before. Following the initial response, inflammation becomes chronic in the majority of cases reviewed herein causing the worsening of alterations affecting the vessel wall, the immune, and coagulation system.

Endothelial dysfunction, initially described in uremic patients, has been also reported in a variety of clinical conditions such as cirrhosis, diabetes, liver transplantation, and even major depression [98, 99]. Inflammatory response occurring during sepsis, infection, and IBD leads to an endothelial damage causing a vascular response and enhanced leukocyte and Plts activation. Release and exposure of adhesion molecules (e.g., P-selectin, E-selectin, ICAM-1, and VCAM-1) play a key role in promoting the attachment of leukocytes and Plts to the subendothelium [18, 100]. The previous events and the presence of collagen and TF on the damaged endothelium will trigger the coagulation cascade and the formation of occlusive thrombi.

Despite the accepted role of the endothelium and Plts in inflammation, less has been discussed on the possible involvement of Plts-and Plt-derived MPs as a carriers of TF and/or modified FVL and their implication in the development thrombotic complications associated to sepsis, infection, or/and IBD [101]. Circulating MPs have been found significantly increased in patients diagnosed with FVL [19]. Moreover, elevated circulating MPs have been also detected in sepsis, infections, and IBD. This fact supports the idea that Plts and both Plt- and leukocyte-derived MPs may develop a crucial role in the progression of inflammation leading not only the formation of the clot but also becoming a predicting factor of the risk of developing thrombosis [42]. It has been demonstrated that MPs increase vascular and endothelial dysfunction, supporting the idea of MPs carrying FVL becoming an enhancer of the prothrombotic events presented in these patients worsening the clinical management and outcome.

Previous studies revealed that MPs are heterogeneous and highly dependent on the activation mechanism that triggered their production [102]. Furthermore, it has been reported that Plt MPs are carriers of specific receptors such as GPIIb-IIIa and other molecules including P-selectin and more importantly FV [2]. In which proportion FVL is present in circulating MPs in FVL disorder as well as during sepsis, infection, and/or IBD may open a new strategy for the clinical management of these patients. Thus, characterization of MPs in patients diagnosed with FVL as well as in patients presenting sepsis, infection, and IBD events may provide valuable information for a better understanding of these disorders and also for the evaluation of the clinical course of these specific conditions. In accordance with the later concept, detection of elevated levels of TF-bearing MVs associated with inflammatory conditions would help to explain the increased risk of thrombosis associated with infections such as IBD [45].

The higher number of MPs loaded with proinflammatory components (FV and TF) leads to a procoagulant state which may be more intense in patients with FVL developing the inflammatory episodes described in this paper [41]. Taking into consideration all the findings reviewed above, it seems reasonable to consider that patients diagnosed with FVL will be exposed to enhanced inflammatory responses in sepsis, infection, or IBD not only due to the FVL itself but also for the high presence of MPs with procoagulant properties. Moreover, recent findings showed that MPs not only are considered vectors of inflammation but are also described as the main responsible of major organ failure in sepsis [103, 104].

Platelet FV has demonstrated to be more resistant to APC action and seems to be the major responsible to prolong the thrombin action once the coagulation cascade is activated [40]. Interestingly, all studies performed trying to elucidate the relationship of FVL with the outcome of these events have been focused on the whole pool of FV. Less has been said about the platelet-derived FV which represents the 20% of the complete pool. Since platelet-derived FV seems to have a major resistance to APC inactivation, it may be reasonable to think about a key role in prolonging the coagulation response and the fact that this effect may be enhanced when FVL mutation occurs.

There is considerable debate on whether FVL could be considered a condition which may lead to an enhanced inflammatory response in different clinical inflammatory conditions. Through the information reviewed herein, it seems that FVL seems not to determine the incidence of these episodes, although there is a common consensus on FVL being considered an important risk factor for the development of thrombotic events in patients diagnosed with sepsis, infection, or IBD. There is a common agreement that homozygous patients for FVL will develop thrombotic episodes during the course of such inflammatory conditions. It is important to take into consideration that heterozygousness for the mutation usually present low risk of developing thrombotic complications under physiological conditions. The risk of thrombosis could increase during an inflammatory response. The presence of the mutation (even in its heterozygous version) during an inflammatory episode may increase the risk of developing abnormal clots and complicate the normal resolution of the episode or its medical treatment.

## 6. Conclusion

Patients with FVL present a thrombophilic tendency that may be enhanced during an inflammatory episode occurring during sepsis, infection, or IBD. Damage of the endothelium, exposure of adhesion molecules, and the participation of procoagulant components involved in inflammation with activation of leukocytes and Plts as well as increased levels of Plts-derived MPs carrying TF and FVL play a key role in upregulating the underlying thrombophilic state even in patients with the heterozygous FVL mutation.

## Figures and Tables

**Table 1:** Genetic risks factor in venous thromboembolism (adapted from [38]).

Genetic risk	Defficiency or alteration	Frequency in patients with thrombosis
Strong genetic risk	Antithrombin	2–5%
	Protein C	2–5%
	Protein S	2–5%
Moderate genetic risk	**Factor V Leiden**	**18**–**35%**
	Prothrombin G20210A	7–10%
	Homocystinemia	18–20%
	Dysfibrinogehemia	10%
Variable genetic risk	Elevated levels FI. FVIII, FIX, FXI	15–30%
Acquired condition	Antiphospholipid antibody/syndrome	5–10%
	Pregnancy	—
	Cancer	—

## Abbreviations

APC: Activated protein C
DVT: Deep venous thrombosis
FV: Factor V
FVL: Factor V Leiden
IBD: Inflammatory bowel disease
ICAM-1: Intercellular adhesion molecule 1
PECAM-1: Platelet-endothelial cell adhesion molecule 1
PSLG-1: P-selectin glycoprotein ligand-1
Plts: Platelets
MPs: Microparticles
MVs: Microvesicles
SIR: Systemic inflammatory response
TF: Tissue factor
VCAM-1: Vascular cell adhesion protein 1
VTE: Venous thromboembolism
VT: Venous thrombosis
vWF: Von Willebrand factor.
